# Systemic injection of nicotinic acetylcholine receptor antagonist mecamylamine affects licking, eyelid size, and locomotor and autonomic activities but not temporal prediction in male mice

**DOI:** 10.1186/s13041-022-00959-y

**Published:** 2022-09-06

**Authors:** Shohei Kaneko, Yasuyuki Niki, Kota Yamada, Daiki Nasukawa, Yusuke Ujihara, Koji Toda

**Affiliations:** 1 Department of Psychology, Keio University, Mita 2-15-45, Minato-ku, Tokyo, Japan; 2 Japan Society for Promotion of Science, Tokyo, Japan; 3 Department of Anatomy and Neurobiology, University of Tennessee Health Science Center, TN Memphis, USA

**Keywords:** Timing and time perception, Learning and memory, Eyelid size, Mecamylamine, Nicotinic acetylcholine receptor, Temporal conditioning, Head-fixed, Mice

## Abstract

Nicotinic acetylcholine receptors are thought to be associated with a wide range of phenomena, such as movement, learning, memory, attention, and addiction. However, the causal relationship between nicotinic receptor activity and behavior remains unclear. Contrary to the studies that examined the functions of muscarinic acetylcholine receptors, the role of the nicotinic acetylcholine receptors on behavior has not been examined as extensively. Here, we examined the effects of intraperitoneal injection of mecamylamine, a nicotinic acetylcholine receptor antagonist, on the performance of male mice in a head-fixed temporal conditioning task and a free-moving open-field task. The head-fixed experimental setup allowed us to record and precisely quantify the licking response while the mice performed the behavioral task with no external cues. In addition, by combining the utility of the head-fixed experimental design with computer vision analysis based on deep learning algorithms, we succeeded in quantifying the eyelid size of awake mice. In the temporal conditioning task, we delivered a 10% sucrose solution every 10 s using a blunt-tipped needle placed within the licking distance of the mice. After the training, the mice showed increased anticipatory licking toward the timing of sucrose delivery, suggesting that the mice could predict the timing of the reward. Systemic injection of mecamylamine decreased licking behavior and caused eye closure but had no effect on learned conditioned predictive behavior in the head-fixed temporal conditioning task. In addition, the injection of mecamylamine decreased spontaneous locomotor activity in a dose-dependent manner in the free-moving open-field task. The results in the open-field experiments further revealed that the effect of mecamylamine on fecal output and urination, suggesting the effects on autonomic activities. Our achievement of successful eyelid size recording has potential as a useful approach in initial screening for drug discovery. Our study paves a way forward to understanding the role of nicotinic acetylcholine receptors on learning and behavior.

**Supplementary Information:**

The online version contains supplementary material available at 10.1186/s13041-022-00959-y.

## Introduction

Nicotinic acetylcholine receptors are ionotropic receptors that respond to the neurotransmitter, acetylcholine. Nicotinic receptors are located in the central nervous system, autonomic ganglion, motor nerve terminals, and other tissues of many organisms, and have been associated with a wide range of behavioral symptoms [1, 2]. Since nicotinic receptors respond to the agonist nicotine, research has primarily focused on their role in addiction [3–5]. Neurological disorders such as Alzheimer’s disease and impairment of learning and memory are related to dysregulation of the acetylcholine system, including muscarinic and nicotinic receptors [6–8]. Although the majority of research efforts on mnemonic functions over the past decades have been devoted to the investigation of signaling through muscarinic subtype cholinergic receptors, the involvement of nicotinic receptors has also been reported [9]. Nicotinic receptor binding is known to be reduced in the cortex, hippocampus [10–12], and striatum [13–15] of Parkinson’s disease patients. Substantial studies have indicated the possibility of manipulating nicotinic receptors in the treatment of psychiatric diseases, such as depression and anxiety [16, 17].

Contrary to studies that examined the role of muscarinic acetylcholine receptors on mnemonic functions, the effects of nicotinic acetylcholine receptor blockade have not been examined extensively. Accumulating evidence suggests that nicotinic acetylcholine receptors may play important roles in learning and memory. Mecamylamine, a ganglionic blocker that has been used as an antihypertensive drug (Inversine®), is a non-competitive antagonist of several nicotinic acetylcholine receptors, including the α4β2 receptor [18]. The α4β2 receptor subtype, the major nicotinic receptors in the brain, is known to be expressed in a wide ranges of brain regions that are related to learning and memory, including cortex, striatum, hippocampus, and amygdala [1, 2]. Mecamylamine impairs the effects of nicotine administration in the peripheral and central nervous systems [18]. In rodents, non-human primates, and humans, mecamylamine has been shown to disrupt learning and memory, such as acquisition and maintenance of spatial information in the Morris water maze and avoidance learning [19–22], attentional and impulsive aspect of learning and behavior in the five-choice serial reaction time task [23, 24], and goal-directed and non-spatial aspect of acquisition and maintenance of learning in the visual discrimination task [25, 26]. These anatomical and pharmacological findings suggest that nicotinic cholinergic activity might be related to learning and memory.

Despite the possible role of cholinergic receptors in learning and memory, the causal relationship between nicotinic cholinergic receptors and temporal prediction remains unclear. Time perception is unique because there are no organs or receptors dedicated to this activity. To understand the neurobiological mechanism of temporal processing, recording physiological measurements while the subjects perform the temporal conditioning task could be helpful. Recently, we developed a novel head-fixed temporal conditioning task [27, 28]. Although the head-fixed temporal conditioning task does not require external sensory cues, whether the mice actually do not use sensory information such as the visual cue was unclear. Head-fixed experimental setup enabled us to record various physiological changes such as the eyelid size while the mice performed the temporal conditioning task. In previous studies that examined the role of nicotinic acetylcholine receptors in free-moving behavior, changes in performance might reflect sensory and motor deficits, instead of changes in learning and memory. Here, we examined the effects of mecamylamine, a nicotinic acetylcholine receptor antagonist, on the performance of mice in a head-fixed temporal conditioning task. In addition, we validated the effects of mecamylamine injection on the whole-body movement by recording locomotor activities in a free-moving open-field task. By comparing the results obtained from the head-fixed temporal conditioning and the open-field tasks, we could examine the relationship between movement and time perception. Measuring the amount of feces and urine after the mice performed the open-field task enabled us to evaluate the effects of mecamylamine on the activity of the autonomic nervous system.

## Results

### Experiment 1: fixed-time schedule task in head-fixed mice

**Fig. 1:**
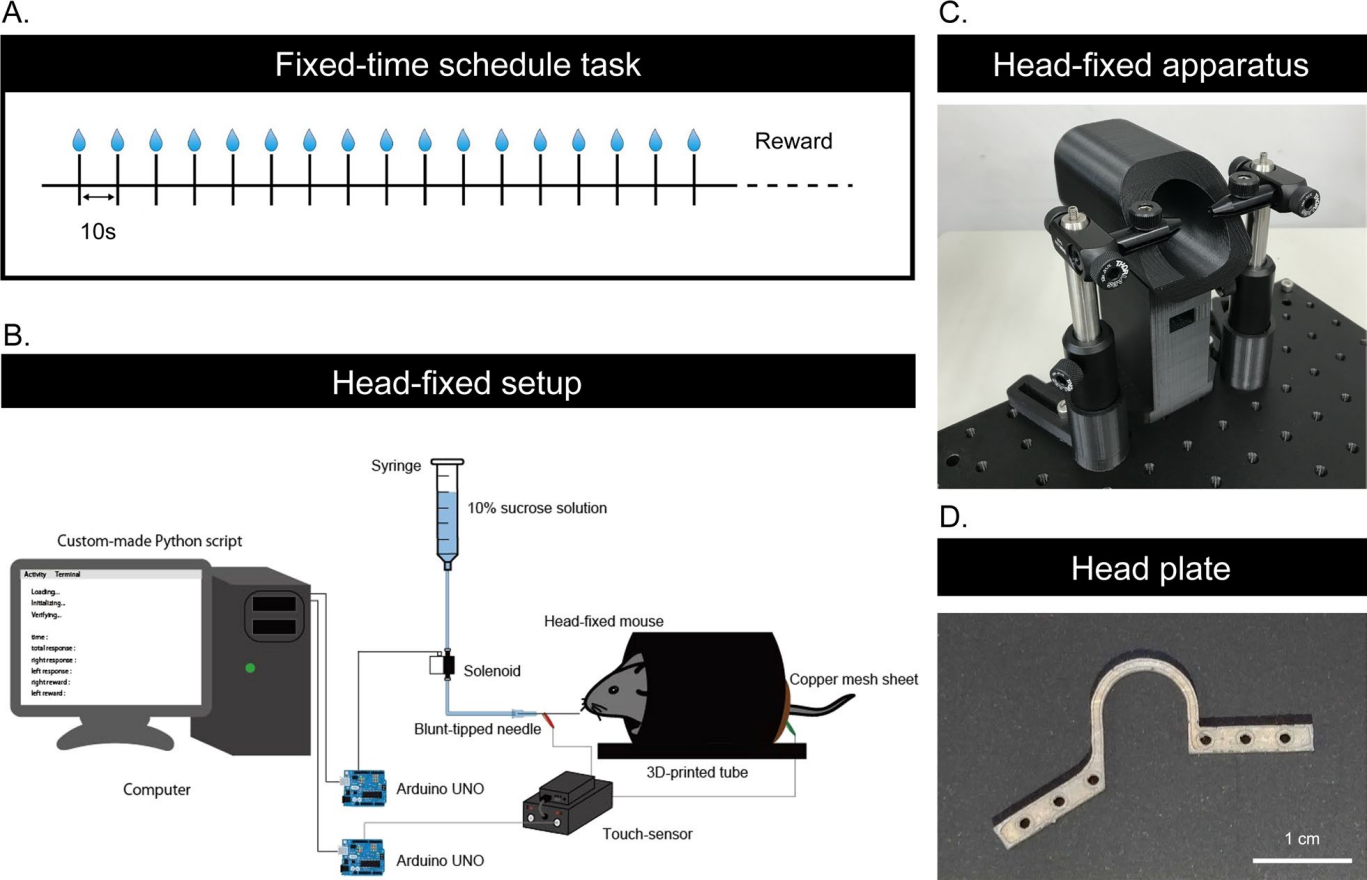
Temporal conditioning task. **A** Graphical representation of the temporal conditioning task. Approximately 2 µL of 10% sucrose solution was delivered through the tube every 10 s. **B** Graphical representation of the experimental setup. Head-fixed mice were allowed to voluntarily lick the spout. **C** Picture of the head-fixed apparatus. **D** Picture of the head plate

**Fig. 2:**
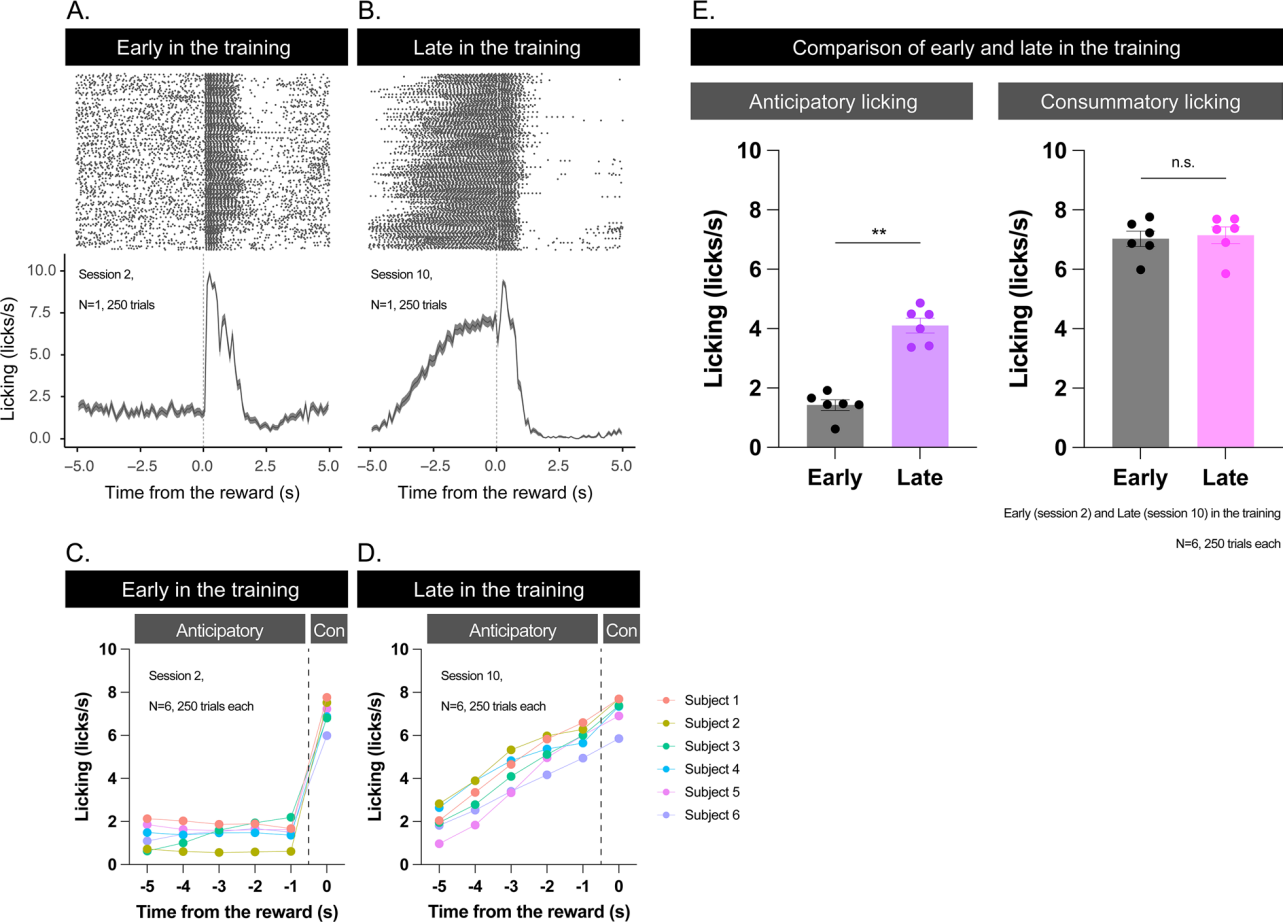
Licking responses in early and late stages of training in the head-fixed temporal conditioning task. **A** Example of the performance of a mouse in early session of the training (session 2; *N* = 1, 250 trials). **B** Example of the performance of a mouse in late session of the training (session 10; *N* = 1, 250 trials). Top: Raster plot of licking responses in a session. The vertical axis indicates the number of trials, and the horizontal axis indicates the time from the reward delivery. Bottom: Histogram of the licking responses in a session. The vertical axis indicates the licks per second and the horizontal axis indicates the time from the reward delivery. **C** Summary of the performance of mice in early session of the training (session 2; *N* = 6, 250 trials each). “Anticipatory” indicates anticipatory licking. “Con” indicates consummatory licking. **D** Summary of the performance of mice in late session of the training (session 10; *N* = 6, 250 trials each). “Anticipatory” indicates anticipatory licking. “Con” indicates consummatory licking. **E** Comparison of the performance between early and late sessions of the training. Left: Comparison of the performance of anticipatory licking between early and late sessions of the training (*N* = 6, 250 trials each, **p < 0.01). Right: Comparison of the performance of anticipatory licking between early and late sessions of the training (*N* = 6, 250 trials each, “n.s.” indicates not significant). Error bars indicate standard error of the mean

To investigate whether the mice can learn to predict the timing of the reward, we trained the mice on a temporal conditioning task with no external sensory cues to signal the timing of the reward. In this fixed-time schedule task, we delivered a 10% sucrose solution every 10 s with a blunt-tipped needle placed within licking distance of head-fixed mice (Fig. 1). At the beginning of training (second session of the training), the mice did not show an increasing pattern of anticipatory licking (Fig. 2A for the example data and Fig. 2D for the summary; *F* (1.102, 5.512) = 0.5623, *p* = 0.507, repeated measures One-way ANOVA). After the training (tenth session of the training), all mice showed increased anticipatory licking toward the timing of reward delivery (Fig. 2B for the example data and Fig. 2D for the summary; *F* (1.303, 6.517) = 95.35, *p* < 0.0001, repeated measures One-way ANOVA, *p* < 0.05, post-hoc Tukey test between all the pairs). Compared to the beginning of the training, anticipatory licking was increased (Fig. 2C; *t* (5) = 7.311, *p* = 0.0007, paired t-test) but consummatory licking was not increased (Fig. 2D; *t* (5) = 0.7852, *p* = 0.4668, paired t-test) after the training. These results suggest that the mice could predict the timing of the reward.

**Fig. 3:**
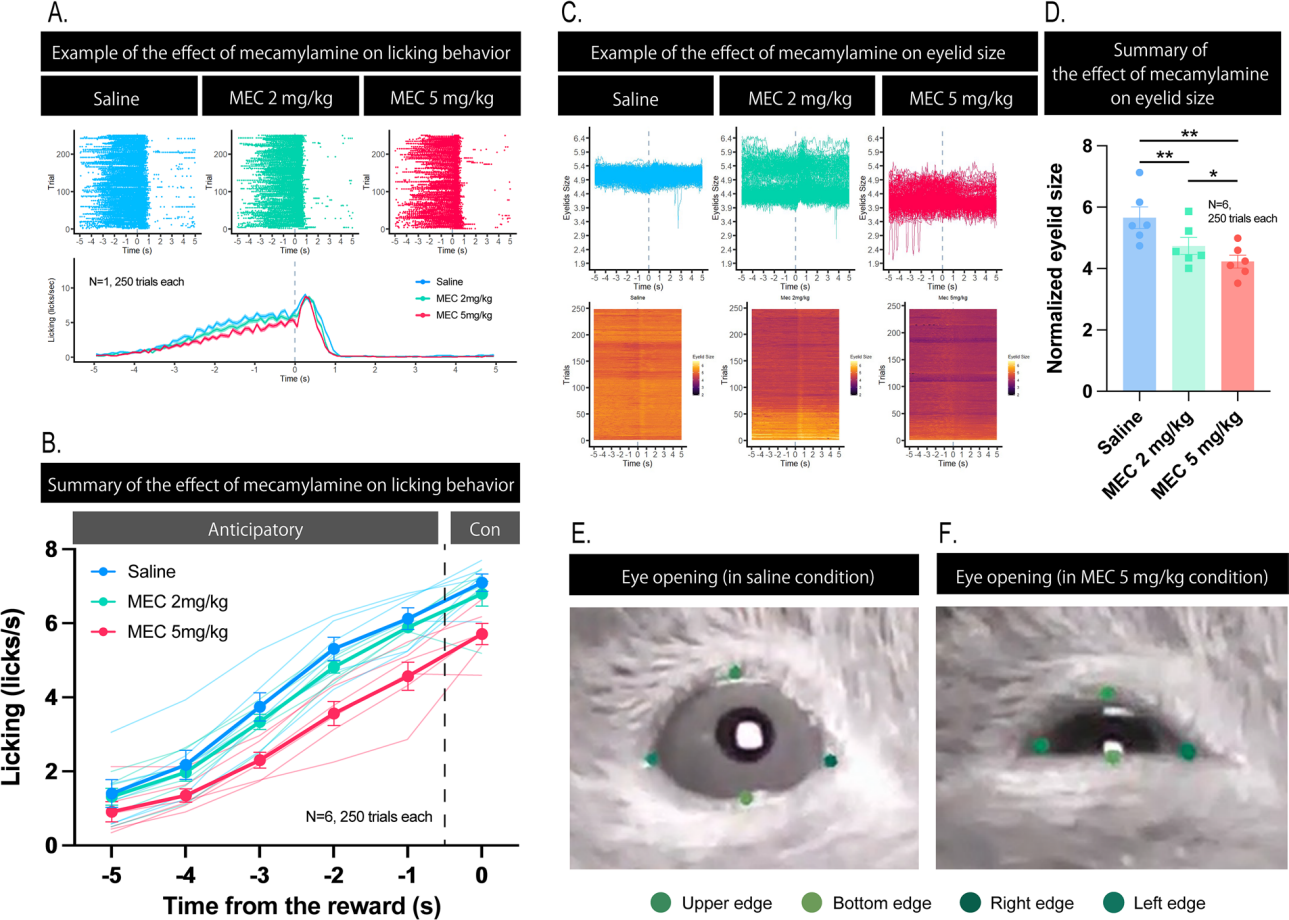
Effects of mecamylamine on the performance in the temporal conditioning task. **A** Examples of the effect of mecamylamine on licking responses in a mouse (*N* = 1, 250 trials each). Each color indicates drug conditions. Upper panel shows the raster plots of the licking responses. Bottom plot shows the histogram of the licking responses. The vertical axis indicates the time from the reward delivery. The horizontal axis indicates the frequency of licking. **B** Summary of the effect of mecamylamine on both anticipatory and consummatory licking (*N* = 6, 250 trials each). “Anticipatory” indicates anticipatory licking. “Con” indicates consummatory licking. We defined anticipatory licking as the period from 5 s before the time of sucrose solution delivery to the time of sucrose solution delivery, and consummatory licking as the period from the time of sucrose solution delivery to 1 s after the time of sucrose solution delivery. **C** Examples of the effect of mecamylamine on the eyelid size in a mouse (N = 1, 250 trials each). Top: Individual plot of the eyelid size. Bottom: heatmap of the eyelid size. **D** Summary of the effect of mecamylamine on the eyelid size (*N* = 6, 250 trials; **p < 0.01, *p < 0.05). **E** Examples of eye-opening in a mouse. **F** Examples of eye-closure in a mouse. “MEC” indicates mecamylamine. Error bars indicate standard error of the mean

To examine the role of nicotinic acetylcholine receptors on the performance of the temporal conditioning task, we examined the effects of intraperitoneal injections of the nicotinic acetylcholine receptor antagonist mecamylamine on licking, timing behavior, and eyelid size. Mecamylamine decreased licking in a dose-dependent manner (Fig. 3A). Both anticipatory and consummatory licking were significantly decreased in the high-dose 5 mg/kg condition compared with saline and 2 mg/kg conditions (Fig. 3B; anticipatory licking: *F* (2, 15) = 6.268, *p* = 0.0105, repeated-measures two-way ANOVA: “Drug condition” factor × “Time from the reward” factor; *p* < 0.05, post-hoc Tukey test between the pairs of “saline vs. mecamylamine 5 mg/kg” and “mecamylamine 2 mg/kg vs. mecamylamine 5 mg/kg”; consummatory licking: *F* (1.516, 7.578) = 22.84, *p* = 0.0009, repeated-measures one-way ANOVA; *p* = 0.0083, post-hoc Tukey test between saline and mecamylamine 5 mg/kg, *p* = 0.0059, post-hoc Tukey test between mecamylamine 2 mg/kg). In all conditions, anticipatory licking increased as the timing of the reward delivery approaches (Fig. 3A, B; *F* (1.249, 18.73) = 438.4, *p* < 0.001, repeated-measures two-way ANOVA: “Drug conditions” factor × “Time from the reward” factor; *p* < 0.05, post-hoc Tukey test in between all the pairs of “Time from the reward” factor). This trend suggests that the learned conditioned timing response was maintained even after a high-dose mecamylamine injection.

While the mice performed the fixed-time schedule task, we recorded a movie of the mice in the head-fixed condition. Using DeepLabCut [29], we successfully quantified the size of their eyelid (Fig. 3C–F; Additional file 3 and 4: Movie S1 and S2). The eyelid sizes of the mice during the fixed-time schedule task also decreased in a dose-dependent manner (*F* (1.720, 8.599) = 34.70, *p* = 0.0001, repeated measures one-way ANOVA). Eyelid size was significantly decreased in the low-dose mecamylamine 2 mg/kg group (*p* = 0.0153; post-hoc Tukey test between saline and mecamylamine 2 mg/kg) and high-dose mecamylamine 5 mg/kg group compared to the saline group (Fig. 3E; *p* = 0.0006, post-hoc Tukey test between saline vs. mecamylamine 5 mg/kg; *p* = 0.0481, post-hoc Tukey test between mecamylamine 2 mg/kg vs. mecamylamine 5 mg/kg). To examine the time course of the effects of mecamylamine, we started the experiment 30, 60, 90, and 120 min after the high-dose mecamylamine injection. Overall eyelid size gradually increased with increasing starting time after injection (Additional file 1: Fig. S1; *F* (2.206, 6.619) = 22.33, *p* = 0.0010, repeated measures one-way ANOVA).

### Experiment 2: open-field task in free-moving mice

To examine the effect of intraperitoneal injections of mecamylamine on whole-body motor function irrespective of licking movement, we examined the spontaneous locomotor activities of mice in the open-field box. In this open-field experiment, we also examined the effective durations of the mecamylamine on the behavior, including start and end time of the behavioral effect, regardless of the half-life time of mecamylamine. Thus, we started the open-field experiment immediately after the injection of mecamylamine. Similar to the results of the licking response in the head-fixed temporal conditioning task, the spontaneous locomotor activities of the mice also decreased in a dose-dependent manner (*F* (1.347, 6.734) = 9.871, *p* = 0.0137, repeated measures one-way ANOVA). We found a significant decrease in locomotor activity in the high-dose of mecamylamine 5 mg/kg condition compared to the saline condition (*p* = 0.0435, post-hoc Tukey test). Analysis of the locomotor activities in the time course during the open-field task indicated that the effect of intraperitoneal injections of mecamylamine was observed approximately 10 to 60 min after the injection.

To assess the impact of mecamylamine on autonomic nervous system function, we collected and quantified measured feces and urine after the mice performed the open-field task. The effect of mecamylamine on fecal output was significant (*F* (1.000, 5.000) = 75.29, *p* = 0.0003, repeated measures one-way ANOVA). After mecamylamine injection, no feces were found in the box under both low-and high-dose conditions. This result was completely different and drastic compared with the results obtained under saline conditions. On the other hand, injection of the mecamylamine had no effect on the amount of urine output (*F* (1.866, 9.331) = 0.6120, *p* = 0.5520, repeated-measures one-way ANOVA).

Because 90 min is much longer than the duration of the mecamylamine effect on eyelid size observed in the head-fixed temporal conditioning task and in spontaneous activities in the open-field task (10 to 60 min), there was a possibility that urination occurred within 60 min after the injection. In addition, the urine that appeared early after inserting the mice into the box might have dried. To exclude these possibilities, we conducted an open-field task using six mice with a 30 min duration and collected urine after the experiment. The amount of urine was decreased in a dose-dependent manner by the injection of mecamylamine ( Additional file 2: Fig. S2; *F* (1.585, 7.927) = 5.620, *p* = 0.0350, repeated-measures one-way ANOVA; saline vs. mecamylamine 5 mg/kg: *p* = 0.0486, post-hoc Tukey test).

## Discussion

In this study, we examined the effects of mecamylamine, a nicotinic acetylcholine receptor antagonist, on the performance of mice in a head-fixed temporal conditioning task and a free-moving open-field task. The head-fixed experimental setup allowed us to precisely record the quantified licking response while the mice performed the behavioral task. In addition, by combining the utility of the head-fixed setup with computer vision analysis based on deep learning algorithms [29], we succeeded in quantifying eyelid size. The mice were trained on a temporal conditioning task with no external sensory cues. In this task, we delivered a 10% sucrose solution every 10 s using a blunt-tipped needle placed within the licking distance of the mice. After the training, the mice showed increased anticipatory licking toward the timing of sucrose delivery, suggesting that the mice could predict the timing of the reward [27, 28]. Systemic injection of mecamylamine impaired licking behavior and caused eye closure in a dose-dependent manner, but did not affect learned temporal prediction in the head-fixed temporal conditioning task. In addition, systemic injection of mecamylamine decreased spontaneous locomotor activity and stopped fecal output and urination during the open-field task.

In Experiment 1, we examined the effects of intraperitoneal injection of mecamylamine on the performance of the mice in the head-fixed temporal conditioning task. Mecamylamine decreased licking in a dose-dependent manner. Both anticipatory and consummatory licking were significantly decreased in the high-dose 5 mg/kg condition compared to the saline and low-dose 2 mg/kg conditions. These results suggest that licking movement was impaired by the blockade of the nicotinic receptor. However, both in low (2 mg/kg) and high (5 mg/kg) dose conditions, the mice showed a decreased but not completely impaired licking response and increased anticipatory licking toward the timing of the reward delivery, although mecamylamine caused eye closure. These results suggest that memory of the learned conditioned timing response was maintained even after a high-dose of mecamylamine injection. These results also confirmed that our temporal conditioning task, which uses no external sensory stimuli to indicate the timing of the reward, is not dependent on visual information.

The results obtained from the blockade of the nicotinic acetylcholine receptor by the nicotinic receptor antagonist were clearly different from those observed in previous studies using muscarinic acetylcholine antagonists. In a study that examined the effect of scopolamine, a muscarinic acetylcholine antagonist, on timing behavior, consistent distraction of the accuracy of temporal prediction was reported [30–33]. Further studies are necessary to examine whether the precision and accuracy of the timing are affected by the blockade of the nicotinic acetylcholine receptor with a head-fixed peak procedure [27]. The peak procedure is a widely used interval timing procedure, where animals learn to anticipate rewards that become available after a fixed delay. To test the ability of timing and time perception of the animals, long duration no-reward probe trials that are twice or third times longer than trained duration were inserted among the fixed delay to the reward. After learning, animals usually cluster their responses around the trained reward-availability time [34–36]. A direct comparison between the effect of muscarinic and nicotinic acetylcholine receptor antagonists on animals performing the head-fixed temporal conditioning task could be an important step in understanding the cholinergic influence on timing and time perception.

We found the distinct effect of mecamylamine on eyelid size, especially at the high-dose of 5 mg/kg. Classically, mecamylamine has been used as an antihypertensive drug [18]. The antihypertensive effects of mecamylamine reflect its blockade of impulse transmission at sympathetic ganglia due to competition for　nicotinic receptors and stabilization of postsynaptic membranes against excitation by acetylcholine. This sympathetic ganglionic blockade causes blood vessels to dilate and peripheral blood flow to increase, resulting in a reduction in blood pressure [18]. A possible explanation for eye closure caused by mecamylamine might be decreased autonomic functions. The decreased autonomic functions such as blood pressure might have led to a decrease in blood flow around the retina and closed the eyes of the mice. In fact, mecamylamine is known to have parasympathetic-blocking activity, causing constipation, urinary retention, dryness of the mouth and skin, dilation of the pupils, and loss of visual accommodation in humans [18]. These observations in human clinical evidence support our findings in the present study.

Although the effect of mecamylamine on eyelid size is a basic physiological phenomenon, it has important implications for future research. If the eyes are closed, the performance of a behavioral task that depends on visual information can be critically affected. In fact, injections of mecamylamine impair visual discrimination [23, 24] and five-choice serial reaction time tasks [25, 26] that require visual information. Before interpreting the results of pharmacological and other interventions as effects of learning, memory, attention, and/or other “cognitive” impairments, we need to be cautious when considering that the results are caused by the impairment of basic physiological mechanisms of sensory information.

**Fig. 4:**
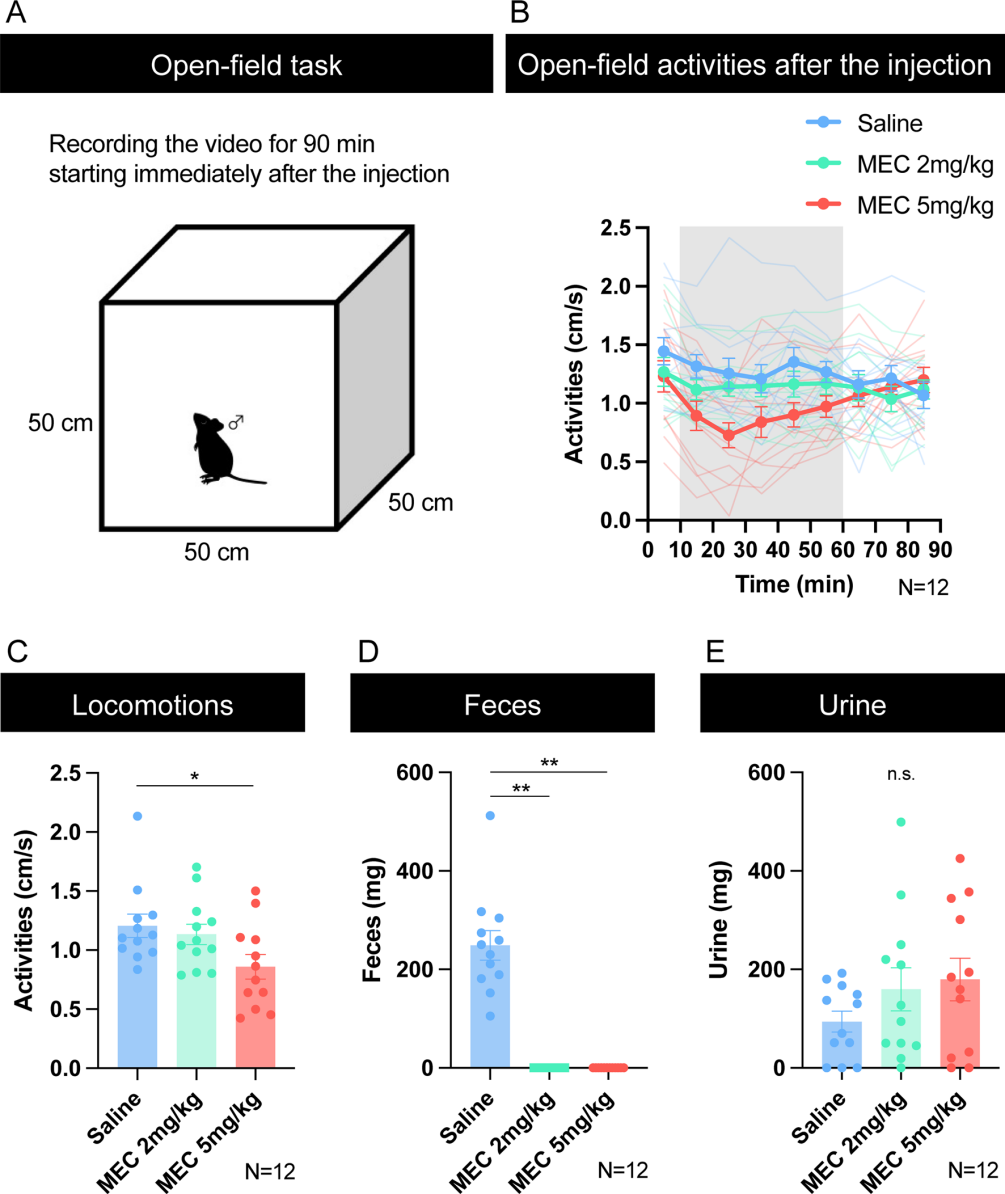
Performance in the open-field task. **A** Open-field task. Habituation to the apparatus and saline injection was performed for 30 min per day for 3 days prior to the start of the experiment. The apparatus was a custom-made white vinyl chloride box measuring 50 cm length × 50 cm width × 50 cm height. Cameras (Logicool HD Webcam C270n, Logicool) were placed overhead, 106 cm from the bottom of the box. Videos were recorded on Windows PC. During habituation, the animals were placed in the open-field box immediately after receiving the intraperitoneal injection of saline and allowed to behave freely. The order of the saline and mecamylamine 2 mg/kg and 5 mg/kg injection was randomized. The duration of the experiment was set at 90 min. A white noise (75 dB) was presented throughout the experiment to mask external noise. **B** Time course of open-field activities after the injection of saline or mecamylamine (*N* = 12). **C** Overall locomotor activities from 10 to 60 minutes from the start of the open-field task (*N* = 12; *p < 0.05). **D** Amount of feces collected after 90 minutes of open-field task (*N* = 12; **p < 0.01). **E** Amount of urine collected after 90 minutes of open-field task (*N* = 12). “MEC” indicates mecamylamine. Error bars indicate standard error of the mean

We could not quantify the pupil size while the mice performed the temporal conditioning task. Because the eyes of mice were closed by the injection of mecamylamine, this effect made it impossible to quantify the pupil size of the mice. However, comparison between Fig. 4E, F clearly shows that the effect of mecamylamine on the pupil size though this is quantitative observation (See also Additional file 3 and 4: Movie S1 and S2). Pupil size is known to reflect attention and arousal of the subjects [37, 38]. Recording the eyelid and the pupil size while the mice perform the behavioral task may pave a new way forward to unweave the intricate relationship between attention, arousal, and time perception.

Quantifying eyelid size was difficult without the head-fixed experimental setup combined with machine vision analysis based on deep learning [29]. Although the technique of recording eyelid size from head-fixed mice has been used in head-fixed eye-blink conditioning experiments [39], we combined this technique with the head-fixed temporal conditioning and pharmacological manipulations. The relationship between autonomic activities and psychiatric diseases, such as anxiety, depression, and insomnia, is evident because the balance between the parasympathetic and sympathetic nervous systems plays a critical role in these phenomena. Our successful eyelid size recording could have potential as a useful approach in initial screening for drug discovery.

In Experiment 2, we examined the effects of intraperitoneal injection of mecamylamine on the performance of the open-field task. The injection of mecamylamine decreased spontaneous locomotor activity in an open-field task in a dose-dependent manner. The effect of mecamylamine was observed after 10–20 min, peaked around 30–40 min, and lasted until 50–60 min after injection. As the total experimental duration in the head-fixed temporal conditioning task was 40–50 min, the duration of the effects of mecamylamine enabled us to compare the results of Experiments 1 and 2. Taken together, these results confirmed that the effect of mecamylamine was not limited to orofacial movement, but also locomotor movement. However, it is not conclusive whether this effect is caused by motor function. As discussed earlier, mecamylamine is a ganglion blocker, so there is a possibility that it might decrease arousal, motivation, or blood pressure and affect locomotor activity.

Another explanation for the decreased licking and locomotor movements after the injection of mecamylamine could be the effect on motor related brain regions in the brain [1, 2]. Because nicotinic acetylcholine receptors are expressed in cortex including motor cortex, striatum, and cerebellum [1, 2], decreased activities of motor related circuits might cause the deficit of licking and locomotor activities. Nicotinic acetylcholine receptors are expressed also in ventral tegmental area and other motivation related areas, decreased motivation might cause the decrease in licking and locomotor movements. Future studies need to dissociate the effects of mecamylamine on movement and motivation.

By collecting feces and urine after the mice performed the open-field task, we verified that injection of mecamylamine drastically inhibited fecal output and urination. These results suggest that the injection of mecamylamine affected the functions of autonomic system. Fecal output during the open-field task has been scored as an index of stress-induced autonomic response [40, 41]. Mecamylamine is a ganglion blocker that disrupts the autonomic nervous system activity. When both sympathetic and parasympathetic activity are inhibited, the effect depends on the contributions of these two systems under normal conditions. In human clinical applications, mecamylamine is known to cause constipation [18]. The injection of mecamylamine completely inhibited fecal output, even at a low dose of 2 mg/kg. Although the effect of mecamylamine on fecal output is distinctive, its critical effect on autonomic activities might be utilized effectively with adequate amounts in future investigations of the use of mecamylamine as a candidate antipsychotic drug for anxiety and depression.

Although we used only male mice for both the head-fixed temporal conditioning and the open-field experiments in this study, accumulating evidence suggest that there are sex differences in expressions of nicotinic receptors [42, 43]. Future studies should include female subjects to understand the way biological sex and sex hormones influence the effects of pharmacological manipulations of nicotinic receptors.

Here, we examined the effects of mecamylamine, a nicotinic acetylcholine receptor antagonist, on the performance of mice during a head-fixed temporal conditioning task and a free-moving open-field task. Contrary to studies that examined the role of muscarinic acetylcholine receptors on learning and behavior, the effects of nicotinic acetylcholine receptor blockade on behavior have not been examined extensively. Although the relationship between nicotinic acetylcholine receptors and Parkinson’s disease implies that nicotinic acetylcholine receptors may play an important role in mnemonic function, we could not find clear evidence that nicotinic acetylcholine receptors play a critical role in the maintenance of learned behavior at least in the appetitive temporal conditioning under the head-fixed condition. Future studies will need to examine other aspects of learning and behavior, such as acquisition and extinction, both in appetitive and aversive conditioning under the head-fixed condition. The head-fixed experimental setup allowed us to precisely record the quantified licking response while the mice performed the behavioral task. In addition, by combining the utility of the head-fixed setup with computer vision analysis based on deep learning algorithms, we succeeded in quantifying eyelid size. Our successful eyelid size recording has potential as a useful approach for initial screening for drug discovery. Taken together, our study paves the way for understanding the role of nicotinic acetylcholine receptors in behavior.

## Methods

### Animals

Eighteen male adult C57BL/6J mice were used in the experiment. All mice were naive at the start of the experiment. Mice were maintained on a reversed 12-h light–dark cycle with lights on at 20:00 and temperature controlled (24 ± 2 °C), in a humidity of 60 ± 20%. We conducted all the experiments between 11:00 and 20:00. For each subject, we tried to run the experiment at the same period of time in the day as long as possible. The experimental and housing protocols adhered to the Japanese National Regulations for Animal Welfare and were approved by the Animal Care and Use Committee of the Keio University.

### Surgery

The mice were anesthetized with 1.0 to 2.5% isoflurane mixed with room air and placed in a stereotactic frame (942WOAE, David Kopf Instruments, Tujunga, CA, USA). A head post (H.E. Parmer Company, Nashville, TN, USA) was implanted on the skull with a dental cement (Product #56,849, 3M Company, Saint Paul, MN, USA) to allow the mice to be head-fixed during the experiment. The mice were group-housed prior to experiments with two to four individuals per cage. After the surgery, the mice were housed singly during recovery for at least two weeks before training began.

### Behavioral tasks

#### Fixed-time schedule task

To investigate whether the mice can learn to predict the timing of the reward, we trained the mice on a temporal conditioning task with no external sensory cues to signal the timing of the reward. Six male mice ranging 4–7 months of age were used in the head-fixed experiment. The mice were water-deprived and received a 10% sucrose solution during the experiments. Their weights were monitored daily. In the head-fixed experiment, we provided the 10% sucrose solution that is enough to maintain over 85% of free-drinking weight of the mice. We also provided additional water provided after the experiment to maintain their weight as needed. The mice had unrestricted access to food in their cages. After recovery from surgery, the mice were water-restricted in their home cage. On the first day of training, the mice were head-fixed briefly and given water rewards to habituate them to the experimental environment. Behavioral experiments were conducted in a square behavioral chamber with a steel drinking spout placed directly in front of the animal’s mouth. Each mouse was kept on a covered elevated platform (custom-designed and 3D printed), with its head fixed by two stabilized clamps holding the sidebars of the head post. The heights of the tunnel and clamps were aligned prior to each session to ensure comfort. The spout and metal sheet under the stage were connected, and individual licking contacts between the mice and the drinking needle were recorded using a contact lickometer. Head-fixed mice were allowed to lick the spout. In the fixed-time schedule task, approximately 2 µL of 10% sucrose solution was delivered through the tube at 10 s intervals (Fig. 1). Sucrose delivery and recording of licking data were conducted using custom-made python3 (version 3.7.7) scripts with a custom-made relay circuit with solenoids. On each day of the experiment, we run one session that contains 250 trials. One trial consists of 10 s interval and the reward delivery. We defined anticipatory licking as the number of licks from − 5 s to 0 s before the reward delivery and consummatory licking as the number of licks from 0 s to + 1 s after the reward delivery.

#### Open-field task

 To examine the effects of mecamylamine on spontaneous locomotor activity, we conducted an open-field task using twelve 2–7 month-old adult male C57BL/6J mice. Habituation to the apparatus and saline injection were performed for 30 min per day for three days prior to the start of the experiment. The apparatus was a custom-made white vinyl chloride box measuring 50 cm in length × 50 cm in width × 50 cm in height. Cameras (Logicool HD Webcam C270n, Logicool) were placed 106 cm above the bottom of the box. The video was recorded on a Windows PC. During habituation, the animals were placed in the open field immediately after receiving the intraperitoneal injection of saline and allowed to behave freely. The order of the saline and mecamylamine 2 mg/kg and 5 mg/kg injections was randomized. Although the half-life of mecamylamine is 1.2 h in rats [44], we set 1–3 days of the interval between each session of the open-field experiments to avoid the effect of mecamylamine injection continues until the next session. The duration of the experiment was 90 min. White noise (75 dB) was presented throughout the experiment to mask the external noise. Every time after running the open-field experiment, we wiped inside the box with 70% alcohol and waited 30 min for drying.

### Recording eyelid size in fixed-time schedule task

An infrared camera was used to capture the eyes of mice during the fixed-time schedule task. The camera was positioned at 45° from the midline of the mouse and 45 mm from the top of the head. Infrared light was used to capture pupils. The brightness of the laboratory was adjusted to 15 lx. DeepLabCut, a tracking tool that uses deep learning, was used to analyze the eye opening [29]. First, we trained DeepLabCut to learn the locations of the upper, lower, left, and right edges of the eyelids, and the trained model was used to analyze the videos and quantify the eyelid size. The median length between the upper and lower eyelids was divided by the median length between the left and right eyelids, and the value multiplied by 10 was quantified as the normalized eyelid size in every frame.

### Drug

We obtained mecamylamine hydrochloride from Cayman Chemical Company (MI, USA). Mecamylamine was dissolved in a saline solution. We administered mecamylamine to mice via intraperitoneal injection at the dose of 10 mL/kg. Two conditions were used for mecamylamine administration: high-dose (5 mg/kg) and low-dose (2 mg/kg). We determined the concentrations of low and high-dose of mecamylamine by referring to existing literature of awake rats [45–47]. The concentrations of 2 mg/kg and 5 mg/kg of mecamylamine are within a range of commonly used dosage (approximately 0.3–10 mg/kg). In the head-fixed temporal conditioning experiment (Experiment 1), the experiments were initiated 5 min after the injection of saline or mecamylamine. In the open-field experiment (Experiment 2), the experiments were initiated soon after the injection of saline or mecamylamine.

### Analysis

RStudio (version 2022.02.0, RStudio PBC, MA, USA) and GraphPad Prism (version 9.3.1, GraphPad, CA, USA) were used for the analysis. We used DeepLabCut [29] to analyze the eyelid size in the head-fixed experiments. In the open-field experiment, we used bonsai [48] to quantify locomotor activity in the box.

## Supplementary Information

**Additional file 1:** **Fig. S1**. Overall eyelid size with different starting time of the experiment after the injection of the mecamylamine. The eyelid size for each subject and the average data are shown. The horizontal axis indicates the conditions after the injection. The vertical axis indicates the normalized eyelid size. Each color indicates an individual subject. The black lines and dots indicate averages. *N* = 4, 250 trials each. Error bars indicate standard error of the mean.

**Additional file 2:** **Fig. S2**. Amount of collectedurine after 30 min of the open-field task. We conducted the open-field task for a 30 min and collected urine after the experiment. The amount of urine was decreased in a dose-dependent manner by the injection of mecamylamine (*F*(1.585, 7.927) = 5.620, *p* = 0.0350, repeated-measures one-way ANOVA; saline v.s. mecamylamine 5 mg/kg: *p =* 0.0486, post-hoc Tukey test). *p < 0.05, *N* = 6. Error bars indicate standard error of the mean.

**Additional file 3:** **Movie S1**. Movie of the eye while the mouse performed the temporal conditioning task after the injection of saline.

**Additional file 4:** **Movie S2**. Movie of the eye while the mouse performed the temporal conditioning task after the injection of mecamylamine 5 mg/kg.

## Data Availability

Data supporting the findings of this study are available from the corresponding author upon reasonable request. The original codes written for the analysis are available from the corresponding author upon reasonable request.
