# Supplementary figures and images for: Systemic injection of nicotinic acetylcholine receptor antagonist mecamylamine affects licking, eyelid size, and locomotor and autonomic activities but not temporal prediction in male mice
Source: Mol Brain. 2022 Sep 6;15:77. doi: 10.1186/s13041-022-00959-y (PMC9450238; doi:10.1186/s13041-022-00959-y)

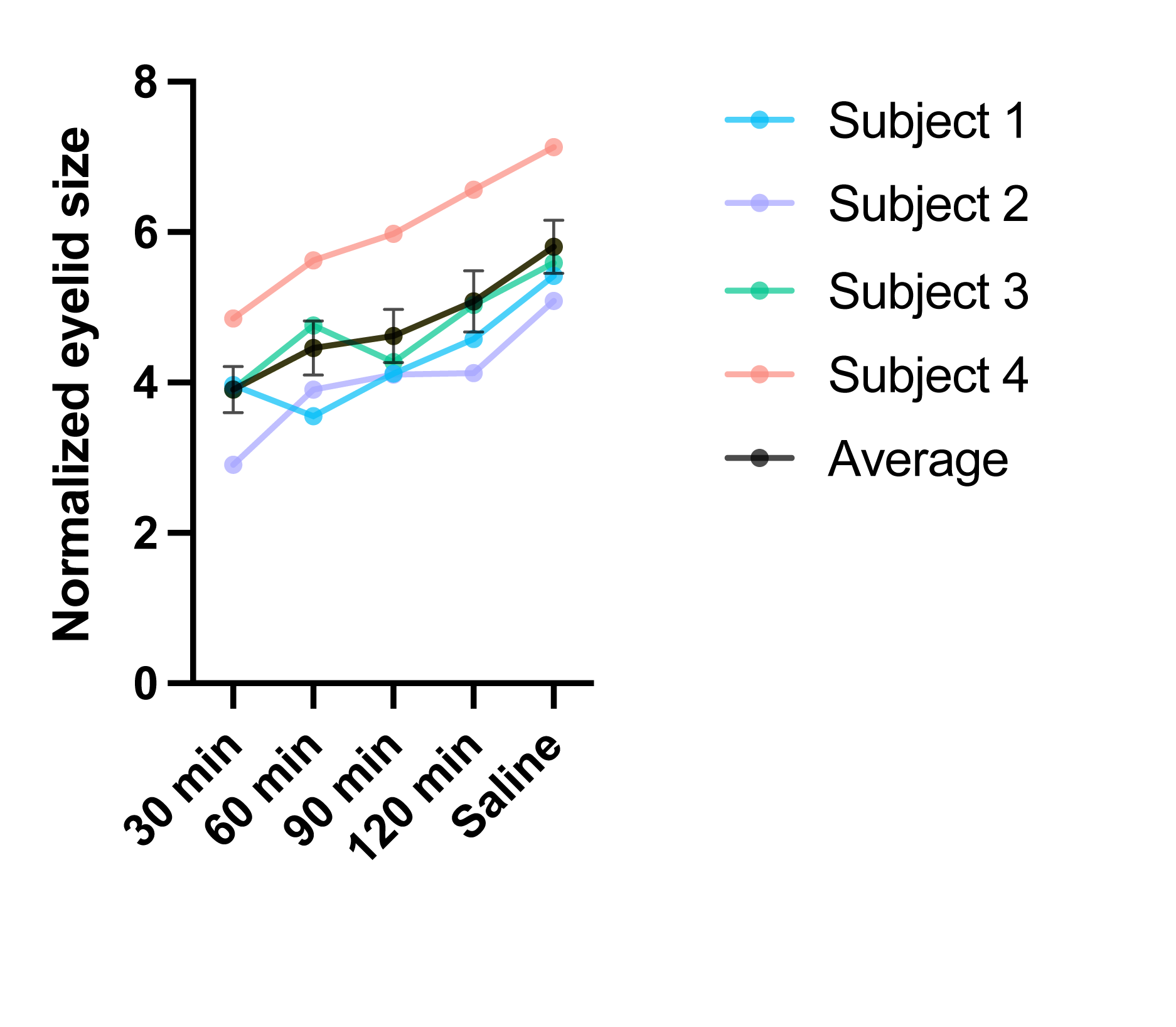

Supplement: Supplementary file 1 — Additional file 1: Fig. S1. Overall eyelid size with different starting time of the experiment after the injection of the mecamylamine. The eyelid size for each subject and the average data are shown. The horizontal axis indicates the conditions after the injection. The vertical axis indicates the normalized eyelid size. Each color indicates an individual subject. The black lines and dots indicate averages. N = 4, 250 trials each. Error bars indicate standard error of the mean. [file 13041_2022_959_MOESM1_ESM.tif]

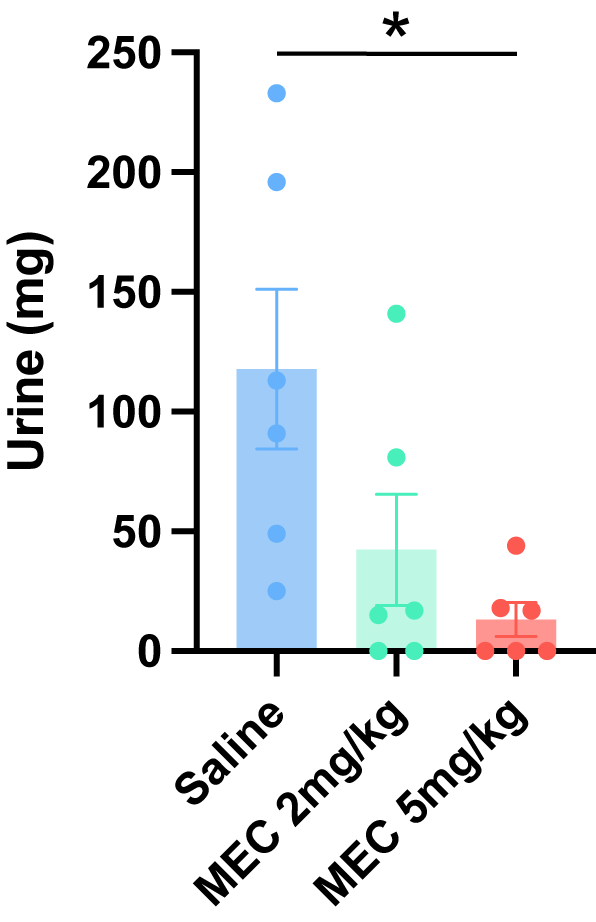

Supplement: Supplementary file 2 — Additional file 2: Fig. S2. Amount of collectedurine after 30 min of the open-field task. We conducted the open-field task for a 30 min and collected urine after the experiment. The amount of urine was decreased in a dose-dependent manner by the injection of mecamylamine (F(1.585, 7.927) = 5.620, p = 0.0350, repeated-measures one-way ANOVA; saline v.s. mecamylamine 5 mg/kg: p = 0.0486, post-hoc Tukey test). *p < 0.05, N = 6. Error bars indicate standard error of the mean. [file 13041_2022_959_MOESM2_ESM.tif]
